# Effect of Hypertension, Waist-to-Height Ratio, and Their Transitions on the Risk of Type 2 Diabetes Mellitus: Analysis from the China Health and Retirement Longitudinal Study

**DOI:** 10.1155/2022/7311950

**Published:** 2022-08-21

**Authors:** Lin Han, Xiaoyan Li, Xin Wang, Jiao Zhou, Qiang Wang, Xiujuan Rong, Gang Wang, Xiaoli Shao

**Affiliations:** ^1^The First Affiliated Hospital of Xi'an Medical University, Xi'an, China; ^2^Heze Medical University, Heze, China

## Abstract

**Background:**

Diabetes is a major reason of death and disability worldwide and frequently coexists with hypertension and central obesity. This study is aimed at investigating the effects of hypertension, waist-to-height ratio (WHtR), and their dynamic transitions on type 2 diabetes mellitus (T2DM) onset among middle-aged and elderly people in China.

**Methods:**

We analyzed 9843 participants free of T2DM (average age, 59.04 ± 9.26 years) at baseline from the China Health and Retirement Longitudinal Study. We classified the participants into the following four categories based on hypertension and WHtR statuses: nonhypertensive with a normal WHtR (NHNW); hypertensive with a normal WHtR (HTNW); nonhypertensive with an elevated WHtR (NHEW); and hypertensive with an elevated WHtR (HTEW). By using a Cox proportional hazards regression model, we assessed whether hypertension, WHtR, and their transitions over time correlated with T2DM risk.

**Results:**

During the follow-up of 8 years, 1263 participants developed incident T2DM. The hazard ratio (HR) for T2DM was 1.48 (95% CI: 1.12, 1.97), 1.56 (95% CI: 1.27, 1.92), and 2.15 (95% CI: 1.74, 2.67) in the HTNW, NHEW, and HTEW groups, respectively, compared with the NHNW group after controlling for confounding factors. When stratified by statuses of hypertension and WHtR transitions, the participants who transitioned from HTNW to HTEW (HR = 1.98, 95% CI: 1.24-3.17), or NHEW to NHNW/HTNW (HR = 1.74, 95% CI: 1.14–2.65), or remained NHEW (HR = 1.42, 95% CI: 1.04-1.93), or NHEW to HTEW (HR = 2.40, 95% CI: 1.66-3.49), or remained HTEW (HR = 2.51, 95% CI: 1.87-3.37) during the follow-up period showed a higher T2DM risk than the consistently NHNW participants.

**Conclusions:**

Being HTNW, NHEW or HTEW or occurring adverse transitions between those states was strongly associated with T2DM onset. Effectively warding off hypertension and central obesity or preventing their further aggravation may substantially decrease the T2DM risk.

## 1. Introduction

Diabetes mellitus is a major metabolic disorder with rapidly rising worldwide prevalence. In the 2019 International Diabetes Federation Diabetes Atlas, the number of patients with diabetes was reported to be approximately 463 million and estimated to reach 700 million by 2045 [1]. Among all countries, China has the highest number of patients with diabetes. The prevalence of diabetes in China is approximately 11.6% [2], and over half (53.6%) of adult patients remain undiagnosed [3]. In addition, diabetes and hypertension are the leading causes of death and disability and frequently coexist [4, 5]. Studies have reported that approximately 70% of patients with type 2 diabetes mellitus (T2DM) have hypertension, and concurrence of T2DM and hypertension increases cause-specific as well as all-cause mortality [6, 7]. Moreover, T2DM with hypertension has been regarded as an independent risk factor for severe organ involvement [8]. Growing evidence has demonstrated that hypertension and T2DM usually occurred successively [9], and compared with normotensive individuals, hypertensive individuals were 2.5 times more likely to develop T2DM within 5 years [10].

The pathogenesis of diabetes and hypertension caused by obesity is multifactorial and complex, and this study has been supported by prior literatures [11–13]. Some researches identified that obesity is critical for the development of insulin resistance (IR), which is a strong predisposing condition for T2DM [14, 15]. Body mass index (BMI) has been widely used for the screening of overweight/obese; however, BMI is criticized for its lack of body fat distribution assessment [16, 17]. Previous studies showed that Asians are more prone to abdominal visceral fat accumulation, although they have lower BMI than Westerners [18, 19]. There is more evidence indicating that central obesity, which is measured based on waist circumference (WC), waist-to-height ratio (WHtR), or waist-to-hip ratio (WHR), carries an increased risk of IR, metabolic syndrome, and cardiovascular disease (CVD) [20]. Among these indicators, WHtR has been shown to be a stronger predicator for T2DM, hypertension, and CVD risk components than BMI, WC, and WHR [21, 22]. A meta-analysis established the predictive validity of those adiposity indices for T2DM and indicated that WHtR is the best discriminator in both sexes [23]. Another study among US adults revealed that each standard deviation increase in WHtR was associated with about 2-fold increased risk of T2DM [24]. Moreover, prospective and cross-sectional research have proved a tight connection between hypertension and WHtR [25].

Taken together, WHtR, hypertension, and T2DM are closely related. However, the combined effect of hypertension and obesity on T2DM has not been widely studied. Although several studies have demonstrated a very strong association between hypertension and obesity in Chinese adults with T2DM [26, 27], the effects of the transition patterns of these two conditions on the development of T2DM are rather elusive. Increasing evidence has shown that early intervention of hypertension and obesity can substantially reduce T2DM risk, induce T2DM remission, and even preclude complications [28–30]. Therefore, based on the China Health and Retirement Longitudinal Study (CHARLS), a large-scale national prospective cohort study, we aimed to identify the effects of hypertension and WHtR on T2DM onset. In addition, a subgroup analysis was conducted to assess whether the transitions of hypertension and WHtR affect the risk of developing T2DM.

## 2. Materials and Methods

### 2.1. Study Design and Population

Our study used data from CHARLS, an ongoing nationally representative longitudinal survey of adults aged ≥45 in China, designed by the National School of Development at Peking University. CHARLS used a multistage probability sampling and recruited 17,708 participants from 150 counties/districts within 28 provinces in 2011-2012 (wave 1). This effort was followed by three subsequent waves in 2013 (wave 2), 2015 (wave 3), and 2018 (wave 4). CHARLS has previously been described in detail [31]. All the collected data in CHARLS were recorded via a face-to-face computer-assisted personal interview (CAPI). Anthropometric measurements were made in 2011, 2013, and 2015, and blood samples were collected in 2011 and 2015.

**Figure 1:**
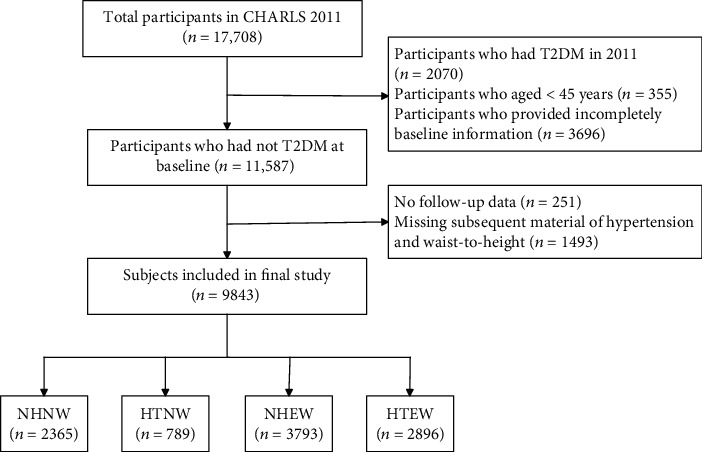
Flowchart of selecting study participants.

We excluded individuals who had baseline T2DM (*n* = 2070); aged <45 years (*n* = 355); or provided incomplete basic socio-demographic, health-related, anthropometric, or biomarker information (*n* = 3696). We further excluded individuals who did not report to the follow-up assessment (*n* = 251) or subsequent material of hypertension and waist-to-height (*n* = 1493). Finally, 9843 individuals who were free of T2DM at baseline and provided complete data required for this study were included. The flowchart describing the exclusion process is illustrated in Figure 1.

### 2.2. Assessment of Hypertension and WHtR

Hypertensive participants were defined as (1) self-reported hypertension diagnosis, and/or (2) a systolic blood pressure (SBP) ≥ 140 mmHg and/or a diastolic blood pressure (DBP) ≥ 90 mmHg, and/or (3) were under antihypertensive medication [32]. Blood pressure was measured three times by trained medical personnel using an Omron HEN-7200 monitor. To determine the blood pressure level, the values from the last two measurements were averaged, excluding the first measurement to avoid white-coat hypertension.

WHtR was calculated by dividing WC (cm) by height (cm). WC was measured by using a nonstretched tape at the navel level at minimal respiration. Height was measured by a Seca™213 stadiometer, with participants standing upright and bare foot on the floor board of the instrument. Anthropometric measurements were performed using standardized procedures. We considered 0.5 as a threshold value of WHtR, and a WHtR above 0.5 was recognized as a risk factor for T2DM [33].

### 2.3. Assessment of T2DM

T2DM was defined as (1) self-reported physician diagnosis, and/or (2) fasting blood glucose (FBG) ≥ 126 mg/dL, and/or (3) glycosylated hemoglobin (HbA1c) ≥ 6.5% [34, 35]. Self-reported data on T2DM was obtained according to the answers to the question “Have you been diagnosed with diabetes by a doctor?”

### 2.4. Operational Definitions

To analyze the effects of hypertension and WHtR on T2DM morbidity, we classified the participants into the following four categories according to hypertension and WHtR statuses: nonhypertensive with a normal WHtR (NHNW); hypertensive with a normal WHtR (HTNW); nonhypertensive with an elevated WHtR (NHEW); and hypertensive with an elevated WHtR (HTEW) (Table 1). Additionally, eleven subgroups from I to XI were defined according to the transition patterns of hypertension and WHtR statuses during the follow-up (Table 2).

### 2.5. Assessment of Covariates

The socio-demographic information about the participants was obtained from standardized questionnaires and included age, gender, hukou (agricultural, nonagricultural, unified residence, or no hukou), residence (rural or urban), education level (illiterate, literate, graduate of primary school, middle school, or high school or more), and marital status (married or others). Health-related variables included self-reported health status (excellent, very good, good, fair, or poor), smoking (yes or no), alcohol drinking (yes or no), and dyslipidemia (yes or no). Smoking status was assessed by asking “Have you smoked >100 cigarettes in your lifetime?” Alcohol consumption was measured by asking “Did you drink any alcoholic beverages in the past year?” Physical examination was conducted by trained medical personnel. BMI was calculated as body mass (kg)/height (m^2^).

Venous blood was drawn for complete blood count (CBC), plasma and buffy coat, and HbA1c assay by the staff of the Chinese Center for Disease Control and Prevention (China CDC) [36]. Dyslipidemia was defined as (1) total cholesterol (TC) ≥ 240 mg/dL, triglycerides (TGs) ≥ 200 mg/dL, low‐density lipoprotein cholesterol (LDL) ≥ 160 mg/dL, or high‐density lipoprotein cholesterol (HDL) < 40 mg/dL and/or (2) self-report of diagnosis with dyslipidemia [37].

### 2.6. Statistical Analysis

The baseline characteristics of the participants were expressed as mean ± standard deviation (SD) for continuous variables and frequencies with percentages for categorical variables. Differences between male and female were compared using the independent *t*-test for continuous variables, and the chi-square (*χ*^2^) test for categorical variables. The follow-up period was from the date of the interview until the date of T2DM diagnosis or the last follow-up. Kaplan-Meier survival curve was used to evaluate the cumulative incidence of T2DM, which was divided into the following groups: NHNW, HTNW, NHEW, and HTEW.

Hazard ratio (HR) for incident T2DM was evaluated for the four categories of hypertension and WHtR statuses by using Cox proportional hazards regression models. The NHNW group of the participants was used as the reference category. Four models were considered to adjust covariates. Model 1 was unadjusted. Model 2 was adjusted for age, hukou, residence, education status, and marital status. Model 3 was additionally adjusted for self-reported health, BMI, smoking, and alcohol consumption. Model 4 adjusted for all the variables in model 3 plus dyslipidemia. All the Cox models were stratified by gender. We also assessed whether there was any association between diabetes and transitions of hypertension and WHtR statuses by using a multivariable Cox proportional hazards regression model. Participants who remained NHNW throughout the follow-up period (group I) were regarded as the reference group.

The statistical analyses were performed using *Stata 15* (*Stata* Corporation, College Station, Texas). All the analyses were two-sided, and a *P* value < 0.05 was considered to indicate statistical significance.

## 3. Results

### 3.1. Participant Characteristics

A total of 9843 participants were included in this study, and their baseline characteristics are shown in Table 3. The mean age was 59.04 (SD: 9.26). Most of the participants had agricultural hukou (83.44%) or were married (87.77%). Approximately two-thirds of the participants lived in rural areas, and approximately 30% of the participants were illiterate and had self-reported good health. The female among the participants had a higher BMI, were less likely to smoke or drink, and were more likely to be NHEW and HTEW than male.

We compared general characteristics of participants included in the study with those excluded from the study because of the incomplete information and T2DM at baseline (Supplementary Table 1 and 2). Population with incomplete data were more likely to be male, nonagricultural hukou, urban residence, and more education when compared to the included population. There was no difference in age, marital status, smoking, and drinking.

### 3.2. Cumulative T2DM Incidence Stratified by Hypertension and WHtR Statuses

**Figure 2:**
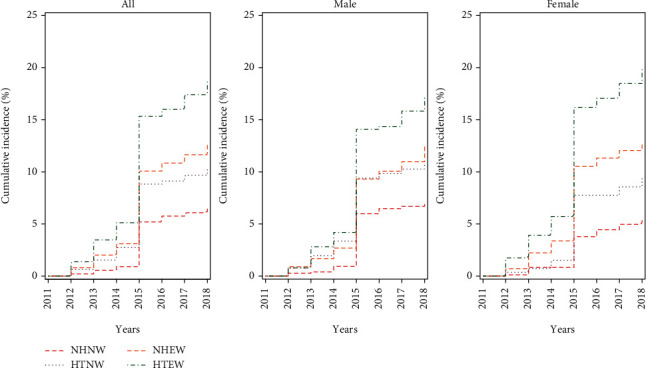
Cumulative Incidence of T2DM according to the four categories of hypertension and WHtR statuses and gender, CHARLS, 2011–2018. Note: NHNW: nonhypertensive with a normal WHtR; HTNW: hypertensive with a normal WHtR; NHEW: nonhypertensive with an elevated WHtR; HTEW: hypertensive with an elevated WHtR.

In CHARLS, 1263 participants, of whom 544 were male, developed T2DM from 2011 to 2018. The cumulative incidence of T2DM was 12.71% among all the participants, and 6.46%, 10.25%, 12.57%, and 18.75% among the NHNW, HTNW, NHEW, and HTEW participants, respectively (Figure 2). Among all the male and female, the cumulative incidence was 11.63% and 13.68%, respectively, and in the NHNW, HTNW, NHEW, and HTEW groups, it was 7.07%, 10.70%, 12.42%, and 17.11%, respectively, for male, and 5.36%, 9.38%, 12.66%, and 19.84%, respectively, for female. The HTEW group was found more likely to develop T2DM than the NHNW, HTNW, or NHEW group, and the female among the participants showed a higher T2DM risk than the male.

### 3.3. Association of T2DM with Hypertension and WHtR Statuses

Table 4 shows the relationship of T2DM onset with hypertension and WHtR statuses among the male and female participants, respectively. The Cox proportional hazards regression analysis showed that the HR for T2DM was 1.63 (95% CI: 1.23, 2.14) for the participants in the HTNW group, 2.00 (95% CI: 1.66, 2.41) for those in the NHEW group, and 3.07 (95% CI: 2.55, 3.69) for those in the HTEW group, compared with the participants in the NHNW group. After adjusting for age, hukou, residence, education, marital status, self-reported health, BMI, smoking, alcohol consumption, and dyslipidemia, the HRs were as follows: 1.48 (95% CI: 1.12, 1.97) for the HTNW group, 1.56 (95% CI: 1.27, 1.92) for the NHEW group, and 2.15 (95% CI: 1.74, 2.67) for the HTEW group. Likewise, subgroup analyses showed that the HR for T2DM was significantly higher among the female than among the male (1.70 and 1.42 among the female and male in the HTNW group, respectively; 1.84 and 1.38 among the female and male in the NHEW group, respectively; and 2.59 and 1.93 among the female and male in the HTEW group, respectively). The association of the confounding factors with T2DM incidence is shown in Supplementary Table 3.

### 3.4. Association of T2DM with Transitions of Hypertension and WHtR

**Figure 3:**
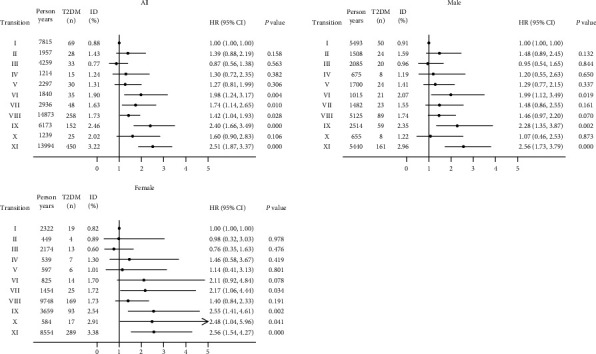
Association of T2DM with hypertension and WHtR transitions in CHARLS 2011–2018. Note: the model was adjusted for age, hukou, residence, education, marital status, self-reported health, BMI, smoking, alcohol consumption, and dyslipidemia. HR: hazard ratio; CI: confidence interval. Subgroup I, stable NHNW; subgroup II, transition from NHNW to HTNW; subgroup III, from NHNW to NHEW; subgroup IV, from NHNW to HTEW; subgroup V, stable HTNW; subgroup VI, from HTNW to HTEW; subgroup VII, from NHEW to NHNW/HTNW; subgroup VIII, stable NHEW; subgroup IX, from NHEW to HTEW; subgroup X, from HTEW to HTNW; subgroup XI, stable HTEW. Subgroup I served as the reference group.

Stratified analyses were performed to assess for the association of hypertension and WHtR transitions with the adjusted HRs of T2DM incidence (Figure 3). Of the eleven transition subgroups, subgroup I consisted of participants who maintained a normal status throughout the follow-up period and served as the reference subgroup. Overall, subgroups VI (HR = 1.98, 95% CI: 1.24-3.17), VII (HR = 1.74, 95% CI: 1.14-2.65), VIII (HR = 1.42, 95% CI: 1.04-1.93), IX (HR = 2.40, 95% CI: 1.66-3.49), and XI (HR = 2.51, 95% CI: 1.87-3.37) were found more likely to develop T2DM than subgroup I. However, the risk for T2MD was not significantly different between subgroup I and subgroups II (HR = 1.39, 95% CI: 0.88-2.19), III (HR = 0.87, 95% CI: 0.56-1.38), IV (HR = 1.30, 95% CI: 0.72-2.35), V (HR = 1.27, 95% CI: 0.81-1.99), and X (HR = 1.60, 95% CI: 0.90-2.83) (Supplementary Table 4). When broken down by gender, similar trends were observed among the male and female participants.

## 4. Discussion

Our study explored the single and joint effects of hypertension and elevated WHtR (≥0.50) on T2DM onset and assessed whether variations in these two conditions had any impact on the risk of T2DM development among middle-aged and elderly Chinese individuals. Compared with the NHNW participants, we observed that the HTNW or NHEW participants had significantly higher risks for T2DM, especially the HTEW participants. This association seemed more evidential in female than their peers. Moreover, analyses stratified by the transition patterns of hypertension and WHtR statuses found that the participants who transitioned from HTNW to HTEW, who transitioned from HNEW to NHNW/HTNW or HTEW, and those who remained NHEW or HTEW had higher T2DM risks than participants who remained NHNW (1.98-, 1.74-, 2.40-, 1.42-, and 2.51-fold higher risks, respectively). However, for participants transitioned from HTEW to NHEW, the risk of T2DM might be not increase during follow-up compared with the persistent NHNW in both sexes.

It is well established that hypertension and central obesity are independent risk factors for T2DM [38, 39]. Here, we further explored the combined effect of hypertension and central obesity. Evidence has suggested that WHtR can serve as a useful anthropometric index for metabolic syndrome [40]. A systematic review and meta-analysis involving >300 000 people from diverse ethnic groups across the world have found that WHtR is superior to other anthropometric indices as an indicator of T2DM [41]. Previous studies have shown that WHtR has a stronger relationship with T2DM prevalence than WC and BMI in the Chinese population [42, 43]. Therefore, in the present study, we used WHtR as the indicator for central obesity.

Hypertension and obesity, individually or concurrently, are two very common comorbidities of T2DM [44]. In this sense, Chaudhary et al. have shown a strong association of hypertension with all adiposity parameters in T2DM patients [39]. Gandotra et al. have reported that in African American adults with obesity, the prevalence of T2DM in hypertensive patients is eight times as high as that in nonhypertensive individuals [45]. In line with these results, our current study revealed that HTEW participants are more influential in glucose abnormalities than HTNW and NHEW patients. These findings were consistent with a previous study conducted in northeast China, which observed the interactive association of hypertension and WHtR with T2DM. The authors of that study found that the risk for T2DM is 3.1-, 2.4-, and 4.9-fold higher in individuals with an elevated WHtR, hypertension, and both conditions, respectively, than in individuals without these conditions [26].

In the cohort study presented here, the cumulative T2DM incidence among the male and female were 11.63% and 13.68%, respectively. We found high prevalence of NHEW and HTEW in females as compared to males, which is more likely to develop T2DM. This result is in line with previous reports in the literature [26, 27]. These gender differences may be partly related to sex hormones, such as estrogen. Sex hormones have been shown to play important roles in the regulation of fat distribution, tissue renin-angiotensin system, and *β*-cell insulin secretion [46–49]. In addition to these intrinsic differences, the changes in the lifestyles of Chinese men and women due to the rapid development of the Chinese economy in recent decades may have caused a considerable impact on the gender differences in hypertension, obesity, T2DM, and CVD. An increasing number of studies suggested that gender differences affect the development and progression of these chronic conditions. A prior study involving adult individuals aged 40-79 from southwest China also confirmed that obesity-related hypertension is more prevalent in female than in male, partly because the female have approximately 40% higher risk for obesity than male [27]. Central obesity, characterized by enlarged fat stores, leads to increased circulating free fatty acids and proinflammatory cytokines [50, 51]. This state can induce peripheral and hepatic insulin resistance, which is a key pathogenic mechanism of diabetes and hypertension [52–54].

Although previous studies have highlighted the influence of hypertension and central obesity on T2DM, only a few studies have investigated the relationship between alterations in blood pressure and abdominal fat with T2DM incidence. We repeated the assessments regarding the effects of hypertension and WHtR on T2DM risk and classified the transition patterns during the follow-up period into eleven subgroups to evaluate the correlation of each pattern with developing T2DM. Our research indicated that the participants who developed to HTEW during follow-up had the highest T2DM incidence than other transitions, especially the persistent HTEW (Figure 3). For populations with hypertension and/or elevated WHtR at baseline, the risk of T2DM is reduced when progress to favorable transitions compared with adverse. Our results suggested that prevention or delay of hypertension and/or elevated WHtR may substantially decrease the T2DM risk. Moreover, the transitions from NHNW to others slightly increased the risk of T2DM compared to consistent NHNW, but the differences were not statistically significant.

Our research has several strengths. This is one of the few prospective cohort studies to examine the associations of hypertension and WHtR transitions with T2DM incidence. We used information from CHARLS, a large nationally representative Chinese study, in which all the participants were ≥45 years of age. Furthermore, all the anthropometric measurements and biochemical tests were performed by trained staff through standardized procedures and rigorous quality controls in CHARLS. However, there are several limitations to be considered. First, our assessments of hypertension and WHtR statuses during the follow-up period relied on measurements of blood pressure, waist circumference, and height every 2 years. The study also lacked a detailed classification of the severity of hypertension and elevated WHtR; thus, our findings may have underestimated the real association. Second, although several possible confounders were taken into account, we cannot exclude unknown confounders, such as dietary patterns and family history of T2DM, due to the scarcity of related data, which may have affected the results. Residual confounding was not completely excluded. Third, the biochemical analysis of blood samples was performed in 2011 and 2015, thus increasing the chance to detect undiagnosed T2DM and leading to a drastic rise in T2DM incidence in 2015.

## 5. Conclusions

This study found that participants with HTNW, NHEW, and HTEW increase the risk of T2DM incidence. The participants who transition to HTEW are closely associated with T2DM onset among middle-aged and elderly Chinese individuals. Accordingly, concurrence of hypertension and central obesity should be considered an independent risk factor for T2DM. Effectively warding off hypertension and central obesity, or prevent their further aggravation, may substantially decrease the T2DM risk.

## Figures and Tables

**Table 1:** The four categories based on hypertension and WHtR statuses.

Hypertension and WHtR status	Definition
NHNW	Nonhypertensive (SBP < 140 mmHg and DBP < 90 mmHg) with a normal WHtR (<0.50)
HTNW	Hypertensive (SBP ≥ 140 mmHg and/or DBP ≥ 90 mmHg) with a normal WHtR (<0.50)
NHEW	Nonhypertensive (SBP < 140 mmHg and DBP < 90 mmHg) with an elevated WHtR (≥0.50)
HTEW	Hypertensive (SBP ≥ 140 mmHg and/or DBP ≥ 90 mmHg) with an elevated WHtR (≥0.50)

**Table 2:** The eleven subgroups based on their transitions during the follow-up.

Transition subgroups	Definition
I	NHNW at baseline, remained stable
II	NHNW at baseline, transitioned to HTNW
III	NHNW at baseline, transitioned to NHEW
IV	NHNW at baseline, transitioned to HTEW
V	HTNW at baseline, remained stable
VI	HTNW at baseline, transitioned to HTEW
VII	NHEW at baseline, transitioned to NHNW/HTNW
Ⅷ	NHEW at baseline, remained stable
IX	NHEW at baseline, transitioned to HTEW
X	HTEW at baseline, transitioned to HTNW
XI	HTEW at baseline, remained stable

**Table 3:** Baseline characteristics of the study participants (CHARLS 2011).

Characteristics	Total	Male	Female	*P* value
Total, *n* (%)	9843	4629 (47.03%)	5214 (52.97%)	—
Age, years	59.04 ± 9.26	59.68 ± 9.09	58.48 ± 9.36	<0.001
BMI, kg/m^2^	23.30 ± 3.82	22.76 ± 3.54	23.77 ± 4.00	<0.001
Hukou, *n* (%)				<0.001
Agricultural	8213 (83.44%)	3780 (81.66%)	4433 (85.02%)	
Nonagricultural	1577 (16.02%)	824 (17.80%)	753 (14.44%)	
Unified residence	52 (0.53%)	25 (0.54%)	27 (0.52%)	
No hukou	1 (0.01%)	0 (0.00%)	1 (0.02%)	
Residence, *n* (%)				0.093
Urban	3305 (33.58%)	1515 (32.73%)	1790 (34.33%)	
Rural	6538 (66.42%)	3114 (67.27%)	3424 (65.67%)	
Education, *n* (%)				<0.001
Illiterate	2805 (28.50%)	601 (12.98%)	2204 (42.27%)	
Literate	1875 (19.05%)	900 (19.44%)	975 (18.70%)	
Primary school	2208 (22.43%)	1286 (27.78%)	922 (17.68%)	
Middle school	1972 (20.03%)	1196 (25.84%)	776 (14.88%)	
High school or more	983 (9.99%)	646 (13.96%)	337 (6.46%)	
Marital status, *n* (%)				<0.001
Married	8639 (87.77%)	4200 (90.73%)	4439 (85.14%)	
Others	1204 (12.23%)	429 (9.27%)	775 (14.86%)	
Self-reported health^∗^, *n* (%)				<0.001
Excellent	304 (3.09%)	180 (3.89%)	124 (2.38%)	
Very good	1204 (12.24%)	658 (14.22%)	546 (10.48%)	
Good	3235 (32.88%)	1593 (34.42%)	1642 (31.50%)	
Fair	3579 (36.37%)	1607 (34.72%)	1972 (37.84%)	
Poor	1518 (15.43%)	590 (12.75%)	928 (17.81%)	
Smoking, *n* (%)				<0.001
Yes	3883 (39.45%)	3442 (74.36%)	441 (8.46%)	
No	5960 (60.55%)	1187 (25.64%)	4773 (91.54%)	
Alcohol drinking, *n* (%)				<0.001
Yes	3250 (33.02%)	2584 (55.82%)	666 (12.77%)	
No	6593 (66.98%)	2045 (44.18%)	4548 (87.23%)	
Hypertension and WHtR status, *n* (%)				<0.001
NHNW	2365 (24.03%)	1520 (32.84%)	845 (16.21%)	
HTNW	789 (8.02%)	516 (11.15%)	273 (5.24%)	
NHEW	3793 (38.53%)	1436 (31.02%)	2357 (45.21%)	
HTEW	2896 (29.42%)	1157 (24.99%)	1739 (33.35%)	
Dyslipidemia, *n* (%)				0.212
Yes	3059 (31.08%)	1410 (30.46%)	1649 (31.63%)	
No	6784 (68.92%)	3219 (69.54%)	3565 (68.37%)	

Note: values were expressed as *n* (%) or mean (SD); BMI: body mass index; NHNW: nonhypertensive with a normal WHtR; HTNW: hypertensive with a normal WHtR; NHEW: nonhypertensive with an elevated WHtR; HTEW: hypertensive with an elevated WHtR. ^∗^The data from some participants were missing.

**Table 4:** Association of T2DM with hypertension and WHtR statuses in CHARLS 2011–2018.

Hypertension and WHtR status	Model 1		Model 2		Model 3		Model 4	
	HR (95% CI)	*P* value	HR (95% CI)	*P* value	HR (95% CI)	*P* value	HR (95% CI)	*P* value
All								
NHNW	1		1		1		1	
HTNW	1.63 (1.23, 2.14)	0.001	1.54 (1.16, 2.05)	0.003	1.51 (1.13, 2.01)	0.005	1.48 (1.12, 1.97)	0.007
NHEW	2.00 (1.66, 2.41)	<0.001	2.06 (1.70, 2.50)	<0.001	1.62 (1.31, 2.00)	<0.001	1.56 (1.27, 1.92)	<0.001
HTEW	3.07 (2.55,3.69)	<0.001	3.15 (2.61, 3.80)	<0.001	2.25 (1.82, 2.78)	<0.001	2.15 (1.74, 2.67)	<0.001
Male								
NHNW	1		1		1		1	
HTNW	1.55 (1.12, 2.17)	0.009	1.45 (1.03, 2.04)	0.031	1.43 (1.02, 2.02)	0.039	1.42 (1.01, 1.99)	0.045
NHEW	1.79 (1.40, 2.29)	<0.001	1.81 (1.41, 2.32)	<0.001	1.43 (1.08, 1.89)	0.012	1.38 (1.04, 1.83)	0.025
HTEW	2.52 (1.98, 3.21)	<0.001	2.61 (2.04, 3.34)	<0.001	1.99 (1.49, 2.66)	<0.001	1.93 (1.43, 2.59)	<0.001
Female								
NHNW	1		1		1		1	
HTNW	1.78 (1.08, 2.93)	0.024	1.83 (1.09, 3.08)	0.022	1.74 (1.03, 2.94)	0.038	1.70 (1.01, 2.88)	0.047
NHEW	2.45 (1.78, 3.38)	<0.001	2.47 (1.79, 3.41)	<0.001	1.88 (1.34, 2.64)	<0.001	1.84 (1.30, 2.58)	<0.001
HTEW	3.95 (2.88, 5.43)	<0.001	4.09 (2.96, 5.66)	<0.001	2.71 (1.91, 3.85)	<0.001	2.59 (1.82, 3.70)	<0.001

Note: Model 1: unadjusted. Model 2: adjusted for age, hukou, residence, education, and marital status. Model 3: adjusted for the variables in model 2 and self-reported health, BMI, smoking, and alcohol consumption. Model 4: adjusted for the variables in model 3 and dyslipidemia. HR: hazard ratio; CI: confidence interval.

## Data Availability

The data used to support the findings of this study are available from the website http://charls.pku.edu.cn/en or corresponding author.

## Supplementary Materials

Supplementary Materials Supplementary Table 1: comparison of baseline characteristics between the participants with complete and incomplete data in CHARLS 2011. Supplementary Table 2: comparison of baseline characteristics between the participants with T2DM and those without T2DM in CHARLS 2011. Supplementary Table 3: associated confounding factors of T2DM incidence, based on a Cox proportional hazards regression model. Supplementary Table 4: characteristics of the eleven transition subgroups.
