# Supplementary material for: Effect of Hypertension, Waist-to-Height Ratio, and Their Transitions on the Risk of Type 2 Diabetes Mellitus: Analysis from the China Health and Retirement Longitudinal Study
Source: J Diabetes Res. 2022 Aug 21;2022:7311950. doi: 10.1155/2022/7311950 (PMC9420619; doi:10.1155/2022/7311950)
Supplement: Supplementary Materials — Supplementary Table 1: comparison of baseline characteristics between the participants with complete and incomplete data in CHARLS 2011. Supplementary Table 2: comparison of baseline characteristics between the participants with T2DM and those without T2DM in CHARLS 2011. Supplementary Table 3: associated confounding factors of T2DM incidence, based on a Cox proportional hazards regression model. Supplementary Table 4: characteristics of the eleven transition subgroups. [file 7311950.f1.docx]

**Effect of Hypertension, Waist-to-height Ratio and Their Transitions on the Risk of Type 2 Diabetes Mellitus: Analysis from the China Health and Retirement Longitudinal Study**

**Supplementary Table 1**: Comparison of baseline characteristics between the participants with complete and incomplete data in CHARLS 2011.

**Supplementary Table 2.** Comparison of baseline characteristics between the participants with T2DM and those without T2DM in CHARLS 2011.

**Supplementary Table 3.** Associated confounding factors of T2DM incidence, based on a Cox proportional hazards regression model.

**Supplementary Table 4.** Characteristics of the eleven transition sub-groups.

**Supplementary Table 1.** Comparison of baseline characteristics between the participants with complete and incomplete data in CHARLS 2011.

| Characteristics | Total Sample | Completed | Incomplete | *P* value |
| --- | --- | --- | --- | --- |
| Total, n (%) | 13539 | 9843 (72.70%) | 3696 (27.30%) | - |
| Age, years | 59.00 ± 9.69 | 59.04 ± 9.26 | 58.89 ± 10.76 | 0.438 |
| BMI*, kg/m^2^ | 23.28 ± 3.87 | 23.30 ± 3.82 | 21.90 ± 6.09 | 0.008 |
| Gender, n (%) |  |  |  | <0.001 |
| Male | 6556 (48.42%) | 4629 (47.03%) | 1927 (52.14%) |  |
| Female | 6983 (51.58%) | 5214 (52.97%) | 1769 (47.86%) |  |
| Hukou, n (%) |  |  |  | <0.001 |
| Agricultural | 10714 (79.13%) | 8213 (83.44%) | 2501 (67.67%) |  |
| Non-Agricultural | 2737 (20.22%) | 1577 (16.02%) | 1160 (31.39%) |  |
| Unified Residence | 82 (0.61%) | 52 (0.53%) | 30 (0.81%) |  |
| No hukou | 6 (0.04%) | 1 (0.01%) | 5 (0.14%) |  |
| Residence, n (%) |  |  |  | <0.001 |
| Urban | 5186 (38.30%) | 3305 (33.58%) | 1881 (50.89%) |  |
| Rural | 8353 (61.70%) | 6538 (66.42%) | 1815 (49.11%) |  |
| Education, n (%) |  |  |  | <0.001 |
| Illiterate | 3696 (27.30%) | 2805 (28.50%) | 891 (24.11%) |  |
| Literate | 2403 (17.75%) | 1875 (19.05%) | 528 (14.29%) |  |
| Primary School | 2972 (21.95%) | 2208 (22.43%) | 764 (20.67%) |  |
| Middle School | 2786 (20.58%) | 1972 (20.03%) | 814 (22.02%) |  |
| High School or more | 1682 (12.42%) | 983 (9.99%) | 699 (18.91%) |  |
| Marital status, n (%) |  |  |  | 0.768 |
| Married | 11876 (87.72%) | 8639 (87.77%) | 3237 (87.58%) |  |
| Others | 1663 (12.28%) | 1204 (12.23%) | 459 (12.42%) |  |
| Self-reported health*, n (%) |  |  |  | <0.001 |
| Excellent | 459 (3.42%) | 304 (3.09%) | 155 (4.33%) |  |
| Very Good | 1728 (12.88%) | 1204 (12.24%) | 524 (14.65%) |  |
| Good | 4371 (32.58%) | 3235 (32.88%) | 1136 (31.76%) |  |
| Fair | 4766 (35.52%) | 3579 (36.37%) | 1187 (33.18%) |  |
| Poor | 2093 (15.60%) | 1518 (15.43%) | 575 (16.07%) |  |
| Smoking, n (%) |  |  |  | 0.760 |
| Yes | 5330 (39.37%) | 3883 (39.45%) | 1447 (39.16%) |  |
| No | 8208 (60.63%) | 5960 (60.55%) | 2248 (60.84%) |  |
| Alcohol drinking, n (%) |  |  |  | 0.175 |
| Yes | 4516 (33.36%) | 3250 (33.02%) | 1266 (34.25%) |  |
| No | 9023 (66.64%) | 6593 (66.98%) | 2430 (65.75%) |  |

Note: Values were expressed as n (%) or mean (SD); BMI, body mass index; *data for some

participants were missing.

**Supplementary Table 2.** Comparison of baseline characteristics between the participants with T2DM and those without T2DM in CHARLS 2011

| Characteristics | Total Sample | T2DM | Without T2DM | *P* value |
| --- | --- | --- | --- | --- |
| Total, n (%) | 11913 | 2070 (17.38%) | 9843 (82.62%) | - |
| Age, years | 59.37 ± 9.37 | 60.92 ± 9.76 | 59.04 ± 9.26 | <0.001 |
| BMI*, kg/m^2^ | 23.51 ± 3.92 | 24.75 ± 4.22 | 23.30 ± 3.82 | <0.001 |
| Gender, n (%) |  |  |  | 0.951 |
| Male | 5602 (47.02%) | 972 (46.96%) | 4629 (47.03%) |  |
| Female | 6311 (52.98%) | 1098 (53.04%) | 5214 (52.97%) |  |
| Hukou, n (%) |  |  |  | <0.001 |
| Agricultural | 9681 (81.26%) | 1468 (70.92%) | 8213 (83.44%) |  |
| Non-Agricultural | 2165(18.17%) | 588 (28.41%) | 1577 (16.02%) |  |
| Unified Residence | 64 (0.54%) | 12 (0.58%) | 52 (0.53%) |  |
| No hukou | 3 (0.03%) | 2 (0.10%) | 1 (0.01%) |  |
| Residence, n (%) |  |  |  | <0.001 |
| Urban | 4297 (36.07%) | 992 (47.92%) | 3305 (33.58%) |  |
| Rural | 7616 (63.93%) | 1078 (52.08%) | 6538 (66.42%) |  |
| Education, n (%) |  |  |  | <0.001 |
| Illiterate | 3353 (28.15%) | 548 (26.47%) | 2805 (28.50%) |  |
| Literate | 2249 (18.88%) | 374 (18.07%) | 1875 (19.05%) |  |
| Primary School | 2654 (22.28%) | 446 (21.55%) | 2208 (22.43%) |  |
| Middle School | 2394 (20.10%) | 422 (20.39%) | 1972 (20.03%) |  |
| High School or more | 1263 (10.60%) | 280 (13.53%) | 983 (9.99%) |  |
| Marital status, n (%) |  |  |  | 0.674 |
| Married | 10449 (87.71%) | 1810 (87.44%) | 8639 (87.77%) |  |
| Others | 1464 (12.29%) | 260 (12.56%) | 1204 (12.23%) |  |
| Self-reported health*, n (%) |  |  |  | <0.001 |
| Excellent | 364 (3.06%) | 60 (2.91%) | 304 (3.09%) |  |
| Very Good | 1393 (11.70%) | 189 (9.17%) | 1204 (12.24%) |  |
| Good | 3770 (31.68%) | 535 (25.95%) | 3235 (32.88%) |  |
| Fair | 4370 (36.72%) | 791 (38.36%) | 3579 (36.37%) |  |
| Poor | 2005 (16.85%) | 487 (23.62%) | 1518 (15.43%) |  |
| Smoking, n (%) |  |  |  | 0.295 |
| Yes | 4674 (39.23%) | 791 (38.21%) | 3883 (39.45%) |  |
| No | 7239 (60.55%) | 1279 (61.79%) | 5960 (60.55%) |  |
| Alcohol drinking, n (%) |  |  |  | 0.002 |
| Yes | 3861 (32.44%) | 611 (29.52%) | 3250 (33.02%) |  |
| No | 8052 (67.59%) | 1459 (70.48%) | 6593 (66.98%) |  |

Note: Values were expressed as n (%) or mean (SD); BMI, body mass index; *data for some

participants were missing.

**Supplementary Table 3.** Associated confounding factors of T2DM incidence, based on a Cox proportional hazards regression model

| Confounders | Hypertension and WHtR Status | | | | *P*^a^ | *P*^b^ | *P*^c^ |
| --- | --- | --- | --- | --- | --- | --- | --- |
|  | NHNW | HTNW | NHEW | HTEW |  |  |  |
| All |  |  |  |  |  |  |  |
| age | 1.00 | 1.02 (1.00, 1.04) | 1.01 (1.00, 1.02) | 1.00 (0.99, 1.01) | 0.007 | 0.007 | <0.001 |
| hukou | 1.00 | 0.83 (0.52, 1.34) | 1.18 (0.92, 1.51) | 1.04 (0.82, 1.31) | 0.453 | 0.183 | 0.760 |
| residence | 1.00 | 1.24 (0.88, 1.76) | 1.39 (1.13, 1.71) | 1.26 (1.04, 1.53) | 0.218 | 0.002 | 0.019 |
| education | 1.00 | 1.02 (0.91, 1.14) | 0.93 (0.87, 1.00) | 1.00 (0.94, 1.07) | 0.744 | 0.052 | 0.963 |
| marital status | 1.00 | 0.87 (0.57, 1.33) | 0.99 (0.75, 1.30) | 1.17 (0.93, 1.47) | 0.508 | 0.922 | 0.190 |
| self-reported health | 1.00 | 0.93 (0.81, 1.06) | 1.05 (0.97, 1.14) | 1.08 (1.00, 1.17) | 0.295 | 0.264 | 0.054 |
| BMI | 1.00 | 1.04 (0.99, 1.10) | 1.06 (1.04, 1.08) | 1.06 (1.04, 1.08) | 0.093 | <0.001 | <0.001 |
| smoking | 1.00 | 0.90 (0.67, 1.20) | 0.88 (0.73, 1.06) | 0.94 (0.79, 1.12) | 0.461 | 0.166 | 0.473 |
| alcohol drinking | 1.00 | 1.10 (0.83, 1.47) | 1.08 (0.89, 1.30) | 1.15 (0.95, 1.38) | 0.502 | 0.449 | 0.160 |
| Dyslipidemia | 1.00 | 0.66 (0.49, 0.88) | 0.70 (0.59, 0.83) | 0.80 (0.69, 0.94) | 0.005 | <0.001 | 0.007 |
| Male |  |  |  |  |  |  |  |
| age | 1.00 | 1.03 (1.01, 1.05) | 1.02 (1.00, 1.03) | 1.00 (0.99, 1.02) | 0.005 | 0.025 | 0.514 |
| hukou | 1.00 | 0.74 (0.43, 1.29) | 1.14 (0.81, 1.60) | 0.88 (0.62, 1.24) | 0.290 | 0.449 | 0.470 |
| residence | 1.00 | 1.11 (0.74, 1.67) | 1.26 (0.94, 1.69) | 1.33 (0.99, 1.77) | 0.608 | 0.125 | 0.057 |
| education | 1.00 | 1.11 (0.96, 1.28) | 1.00 (0.89, 1.11) | 1.07 (0.96, 1.19) | 0.168 | 0.943 | 0.243 |
| marital status | 1.00 | 1.03 (0.62, 1.71) | 0.96 (0.61, 1.51) | 1.18 (0.80, 1.74) | 0.900 | 0.869 | 0.408 |
| self-reported health | 1.00 | 0.91 (0.77, 1.08) | 0.95 (0.85, 1.07) | 0.94 (0.84, 1.06) | 0.277 | 0.437 | 0.316 |
| BMI | 1.00 | 1.03 (0.97, 1.11) | 1.06 (1.03, 1.10) | 1.06 (1.03, 1.09) | 0.266 | <0.001 | <0.001 |
| smoking | 1.00 | 0.98 (0.66, 1.44) | 0.84 (0.64, 1.12) | 0.84 (0.64, 1.11) | 0.903 | 0.242 | 0.230 |
| alcohol drinking | 1.00 | 1.11 (0.81, 1.56) | 1.06 (0.83, 1.35) | 1.16 (0.91, 1.48) | 0.487 | 0.638 | 0.219 |
| Dyslipidemia | 1.00 | 0.68 (0.47, 0.97) | 0.72 (0.56, 0.92) | 0.85 (0.66, 1.08) | 0.033 | 0.010 | 0.186 |
| Female |  |  |  |  |  |  |  |
| age | 1.00 | 1.00 (0.97, 1.03) | 1.01 (1.00, 1.03) | 1.00 (0.98, 1.01) | 0.881 | 0.136 | 0.673 |
| hukou | 1.00 | 1.13 (0.45, 2.83) | 1.22 (0.85, 1.75) | 1.20 (0.87, 1.67) | 0.793 | 0.282 | 0.260 |
| residence | 1.00 | 1.36 (0.68, 2.71) | 1.47 (1.10, 1.97) | 1.21 (0.93, 1.58) | 0.382 | 0.009 | 0.155 |
| education | 1.00 | 0.81 (0.64, 1.02) | 0.89 (0.80, 0.98) | 0.96 (0.88, 1.06) | 0.069 | 0.017 | 0.449 |
| marital status | 1.00 | 0.73 (0.33, 1.63) | 1.03 (0.72, 1.46) | 1.15 (0.86, 1.55) | 0.448 | 0.881 | 0.344 |
| self-reported health | 1.00 | 0.97 (0.76, 1.23) | 1.14 (1.02, 1.28) | 1.21 (1.08, 1.35) | 0.792 | 0.023 | 0.001 |
| BMI | 1.00 | 1.05 (0.97, 1.14) | 1.06 (1.03, 1.09) | 1.07 (1.04, 1.09) | 0.228 | <0.001 | <0.001 |
| smoking | 1.00 | 1.02 (0.46, 2.27) | 0.98 (0.65, 1.48) | 1.08 (0.75, 1.57) | 0.957 | 0.931 | 0.673 |
| alcohol drinking | 1.00 | 1.25 (0.62, 2.53) | 1.17 (0.84, 1.63) | 1.15 (0.80, 1.64) | 0.537 | 0.362 | 0.451 |
| Dyslipidemia | 1.00 | 0.64 (0.37, 1.09) | 0.68 (0.54, 0.86) | 0.78 (0.63, 0.96) | 0.101 | 0.001 | 0.022 |

Note: Data were presented as hazard ratio (95%CI); BMI, body mass index; *P*^a^ for HTNW compared to NHNW; *P*^b^ for NHEW compared to NHNW; *P*^c^ for HTEW compared to NHNW.

**Supplementary Table 4.** Characteristics of the eleven transition sub-groups

| Characteristics | Ⅰ | Ⅱ | Ⅲ | Ⅳ | Ⅴ | Ⅵ | Ⅶ | Ⅷ | Ⅸ | Ⅹ | Ⅺ |  |  |  |  |  |  |  |  |  |  |
| --- | --- | --- | --- | --- | --- | --- | --- | --- | --- | --- | --- | --- | --- | --- | --- | --- | --- | --- | --- | --- | --- |
| Total, n (%) | 1213 | 307 | 656 | 189 | 353 | 300 | 466 | 2343 | 984 | 200 | 2343 |  |  |  |  |  |  |  |  |  |  |
| Age, years | 57.36 ± 8.18 | 61.21 ± 9.45 | 56.64 ± 8.82 | 60.21 ± 9.50 | 62.95 ± 9.27 | 61.95 ± 9.43 | 58.83 ± 8.89 | 56.22 ± 8.20 | 59.49 ± 9.35 | 64.14 ± 9.50 | 61.17 ± 9.45 |  |  |  |  |  |  |  |  |  |  |
| BMI, kg/m2 | 19.80 ± 2.09 | 19.53 ± 2.39 | 21.46 ± 2.45 | 20.88 ± 2.03 | 19.93 ± 2.58 | 21.60 ± 2.89 | 21.91 ± 2.70 | 24.55 ± 3.36 | 24.69 ± 3.27 | 22.40 ± 3.56 | 25.61 ± 3.68 |  |  |  |  |  |  |  |  |  |  |
| Gender, n (%) |  |  |  |  |  |  |  |  |  |  |  |  |  |  |  |  |  |  |  |  |  |
| Male | 855 (70.49%) | 237 (77.20%) | 324 (49.39%) | 104 (55.03%) | 264 (74.79%) | 164 (54.67%) | 232 (49.79%) | 805 (34.36%) | 399 (40.55%) | 100 (50.00%) | 910 (38.84%) |  |  |  |  |  |  |  |  |  |  |
| Female | 358 (29.51%) | 70 (22.80%) | 332 (50.61%) | 85 (44.97%) | 89 (25.21%) | 136 (45.33%) | 234 (50.21%) | 1538 (65.64%) | 585 (59.45%) | 100 (50.00%) | 1433 (61.16%) |  |  |  |  |  |  |  |  |  |  |
| Hukou, n (%) |  |  |  |  |  |  |  |  |  |  |  |  |  |  |  |  |  |  |  |  |  |
| Agricultural | 1070 (88.21%) | 265 (86.32%) | 566 (86.28%) | 164 (86.77%) | 310 (87.82%) | 251 (83.67%) | 397 (85.19%) | 1938 (82.71%) | 807 (82.01%) | 177 (88.50%) | 1856 (79.21%) |  |  |  |  |  |  |  |  |  |  |
| Non-Agricultural | 139 (11.46%) | 42 (13.68%) | 85 (12.96%) | 21 (11.11%) | 40 (11.33%) | 48 (16.00%) | 67 (14.38%) | 390 (16.65%) | 174 (17.68%) | 23 (11.50%) | 473 (20.19%) |  |  |  |  |  |  |  |  |  |  |
| Unified Residence | 4 (0.33%) | 0 | 5 (0.76%) | 4 (2.12%) | 3 (0.85%) | 1 (0.33%) | 2 (0.43%) | 14 (0.60%) | 3 (0.30%) | 0 | 14 (0.60%) |  |  |  |  |  |  |  |  |  |  |
| No hukou | 0 | 0 | 0 | 0 | 0 | 0 | 0 | 1 (0.04%) | 0 | 0 | 0 |  |  |  |  |  |  |  |  |  |  |
| Residence, n (%) |  |  |  |  |  |  |  |  |  |  |  |  |  |  |  |  |  |  |  |  |  |
| Urban | 306 (25.23%) | 70 (22.80%) | 209 (31.86%) | 51 (26.98%) | 73 (20.68%) | 105 (35.00%) | 148 (31.76%) | 824 (35.17%) | 357 (36.28%) | 62 (31.00%) | 923 (39.39%) |  |  |  |  |  |  |  |  |  |  |
| Rural | 907 (74.77%) | 237 (77.20%) | 447 (68.14%) | 138 (73.02%) | 280 (79.32%) | 195 (65.00%) | 318 (68.24%) | 1519 (64.83%) | 627 (63.72%) | 138 (69.00%) | 1420 (60.61%) |  |  |  |  |  |  |  |  |  |  |
| Education, n (%) |  |  |  |  |  |  |  |  |  |  |  |  |  |  |  |  |  |  |  |  |  |
| Illiterate | 259 (21.35%) | 73 (23.78%) | 156 (23.78%) | 67 (35.45%) | 89 (25.21%) | 88 (29.33%) | 132 (28.33%) | 635 (27.10%) | 320 (32.52%) | 74 (37.00%) | 748 (31.92%) |  |  |  |  |  |  |  |  |  |  |
| Literate | 263 (21.68%) | 71 (23.13%) | 129 (19.66%) | 32 (16.93%) | 82 (23.23%) | 53 (17.67%) | 108 (23.18%) | 431 (18.40%) | 170 (17.28%) | 43 (21.50%) | 406 (17.33%) |  |  |  |  |  |  |  |  |  |  |
| Primary School | 275 (22.67%) | 82 (26.71%) | 133 (20.27%) | 40 (21.16%) | 93 (26.35%) | 78 (26.00%) | 97 (20.82%) | 506 (21.60%) | 230 (23.37%) | 47 (23.50%) | 517 (22.07%) |  |  |  |  |  |  |  |  |  |  |
| Middle School | 269 (22.18%) | 57 (18.57%) | 154 (23.48%) | 31 (16.40%) | 61 (17.28%) | 50 (16.67%) | 88 (18.88%) | 512 (21.85%) | 191(19.41%) | 25 (12.50%) | 447 (19.08%) |  |  |  |  |  |  |  |  |  |  |
| High School or more | 147 (12.12%) | 24 (7.82%) | 84 (12.80%) | 19 (10.05%) | 28 (7.93%) | 31 (10.33%) | 41 (8.80%) | 259 (11.05%) | 73 (7.42%) | 11 (5.50%) | 225 (9.60%) |  |  |  |  |  |  |  |  |  |  |
| Marital status, n (%) |  |  |  |  |  |  |  |  |  |  |  |  |  |  |  |  |  |  |  |  |  |
| Married | 1121 (92.42%) | 263 (85.67%) | 589 (89.79%) | 167 (88.36%) | 288 (81.59%) | 244 (81.33%) | 420 (90.13%) | 2134 (91.08%) | 863 (87.70%) | 153 (76.50%) | 1985 (84.72%) |  |  |  |  |  |  |  |  |  |  |
| Others | 92 (7.58%) | 44 (14.33%) | 67 (10.21%) | 22 (11.64%) | 65 (18.41%) | 56 (18.67%) | 46 (9.87%) | 209 (8.92%) | 121 (12.30%) | 47 (23.50%) | 358 (15.28%) |  |  |  |  |  |  |  |  |  |  |
| Self-reported health*, n (%) | | | | | | | | | | | |  |  |  |  |  |  |  |  |  |  |
| Excellent | 30 (2.47%) | 13 (4.23%) | 28 (4.27%) | 8 (4.26%) | 10 (2.83%) | 9 (3.00%) | 11 (2.36%) | 82 (3.50%) | 45 (4.57%) | 5 (2.50%) | 48 (2.05%) |  |  |  |  |  |  |  |  |  |  |
| Very Good | 155 (12.78%) | 41 (13.36%) | 81 (12.35%) | 32 (17.02%) | 40 (11.33%) | 27 (9.00%) | 71 (15.24%) | 321 (13.71%) | 120 (12.20%) | 17 (8.50%) | 239 (10.20%) |  |  |  |  |  |  |  |  |  |  |
| Good | 395 (32.56%) | 102 (33.22%) | 219 (33.38%) | 65 (34.57%) | 99 (28.05%) | 106 (35.33%) | 146 (31.33%) | 807 (34.46%) | 355 (36.08%) | 53 (26.50%) | 729 (31.13%) |  |  |  |  |  |  |  |  |  |  |
| Fair | 451 (37.18%) | 113 (36.81%) | 231 (35.21%) | 59 (31.38%) | 134 (37.96%) | 118 (39.33%) | 163 (34.98%) | 832 (35.53%) | 321 (32.62%) | 78 (39.00%) | 896 (38.26%) |  |  |  |  |  |  |  |  |  |  |
| Poor | 182 (15.00%) | 38 (12.38%) | 97 (14.79%) | 24 (12.77%) | 70 (19.83%) | 40 (13.33%) | 75 (16.09%) | 300 (12.81%) | 143 (14.53%) | 47 (23.50%) | 430 (18.36%) |  |  |  |  |  |  |  |  |  |  |
| Smoking, n (%) |  |  |  |  |  |  |  |  |  |  |  |  |  |  |  |  |  |  |  |  |  |
| Yes | 715 (58.94%) | 204 (66.45%) | 277 (42.23%) | 82 (43.39%) | 237 (67.14%) | 136 (45.33%) | 183 (39.27%) | 652 (27.83%) | 336 (34.15%) | 91 (45.50%) | 781 (33.33%) |  |  |  |  |  |  |  |  |  |  |
| No | 498 (41.06%) | 103 (33.55%) | 379 (57.77%) | 107 (56.61%) | 116 (32.86%) | 164 (54.67%) | 283 (60.73%) | 1691 (72.17%) | 648 (65.85%) | 109 (54.50%) | 1562 (66.67%) |  |  |  |  |  |  |  |  |  |  |
| Alcohol drinking, n (%) | |  |  |  |  |  |  |  |  |  |  |  |  |  |  |  |  |  |  |  |  |
| Yes | 547 (45.09%) | 143 (46.58%) | 243 (37.04%) | 67 (35.45%) | 162 (45.89%) | 118 (39.33%) | 162 (34.76%) | 640 (27.32%) | 304 (30.89%) | 74 (37.00%) | 641 (27.36%) |  |  |  |  |  |  |  |  |  |  |
| No | 666 (54.91%) | 164 (53.42%) | 413 (62.96%) | 122 (64.55%) | 191 (54.11%) | 182 (60.67%) | 304 (65.24%0 | 1703 (72.68%) | 680 (69.11%) | 126 (63.00%) | 1702 (72.64%) |  |  |  |  |  |  |  |  |  |  |
| Dyslipidemia, n (%) |  |  |  |  |  |  |  |  |  |  |  |  |  |  |  |  |  |  |  |  |  |
| Yes | 230 (18.96%) | 48 (15.64%) | 141 (21.49%) | 39 (20.63%) | 81 (22.95%) | 84 (28.00%) | 97 (20.82%) | 763 (32.57%) | 358 (36.38%) | 56 (28.00%) | 1011 (43.15%) |  |  |  |  |  |  |  |  |  |  |
| No | 983 (81.04%) | 259 (84.36%) | 515 (78.51%) | 150 (79.37%) | 272 (77.05%) | 216 (72.00%) | 369 (79.18%) | 1580 (67.43%) | 626 (63.62%) | 144 (72.00%) | 1332 (56.85%) |  |  |  |  |  |  |  |  |  |  |

Note: Values were expressed as n (%) or mean (SD); BMI, body mass index; *data for some participants were missing. HR: hazard ratio. CI: confidence interval. Sub-group I, stable NHNW; sub-group Ⅱ, transition from NHNW to HTNW; sub-group Ⅲ, from NHNW to NHEW; sub-group Ⅳ, from NHNW to HTEW; sub-group Ⅴ, stable HTNW; sub-group Ⅵ, from HTNW to HTEW; sub-group Ⅶ, from NHEW to NHNW/HTNW; sub-group Ⅷ, stable NHEW; sub-group Ⅸ, from NHEW to HTEW; sub-group Ⅹ, from HTEW to HTNW; sub-group Ⅺ, stable HTEW. Sub-group I served as the reference group.
